# Role of Cytosolic Carboxypeptidase 5 in Neuronal Survival and Spermatogenesis

**DOI:** 10.1038/srep41428

**Published:** 2017-01-27

**Authors:** Hui-Yuan Wu, Peng Wei, James I. Morgan

**Affiliations:** 1 Department of Developmental Neurobiology, St. Jude Children’s Research Hospital, Memphis, TN, USA

## Abstract

Proteins may undergo a type of posttranslational modification – polyglutamylation, where a glutamate residue is enzymatically linked to the γ-carboxyl group of a glutamate in the primary sequence of proteins and additional glutamates are then sequentially added via α-carboxyl–linkages to the growing glutamate side chain. Nna1 (a.k.a. CCP1) defines the 6-member cytosolic carboxypeptidase (CCP) family that metabolizes polyglutamate side chain and its loss results in neurodegeneration and male infertility. Whereas most CCPs catalyze hydrolysis of α-carboxyl-linked glutamates, CCP5 uniquely metabolizes the γ-carboxyl linked, branch point glutamate. Using purified recombinant mouse CCP5, we confirmed that it metabolized γ-carboxyl-linked glutamate of synthetic substrates and tubulin. Despite this unique feature and its indispensible functions in lower species, we found that unlike Nna1, CCP5 is not essential for neuronal survival in mouse. CCP5 deficiency does cause male infertility. However, the mechanism by which this occurs is distinct from that of Nna1 loss. Instead, it is phenotypically reminiscent of the infertility of *olt* mice. Our findings suggest that Nna1 and CCP5 do not work coordinately in the same pathway in either the nervous system or spermatogenesis. This is the first study addressing the function of CCP5 in mammals.

The cytosolic carboxypeptidase subfamily of glutamylases (CCP1-CCP6) is involved in a form of posttranslational modification termed protein polyglutamylation^1^,^2^,^3^,^4^. In this process, a glutamate residue is enzymatically linked to the γ-carboxyl group of a glutamate in the gene-encoded sequence of protein substrates and additional glutamates are then sequentially added via α-carboxyl–linkages to the growing glutamate side chain. The formation of polyglutamate chains is catalyzed by the tubulin tyrosine ligase-like (TTLL) family of enzymes^5^,^6^. In contrast, the CCPs are uniquely responsible for degrading polyglutamate chains^1^,^2^,^3^,^4^. Although all CCPs catabolize the polyglutamate side chains of tubulin – the best studied substrate for this form of posttranslational modification^7^,^8^,^9^,^10^, they can be discriminated to some degree based on their enzyme kinetics and preferences for synthetic substrates^4^, suggesting they may not be biologically equivalent. However, until recently little was known of the biological processes in which CCPs or indeed polyglutamylation in general played a crucial role.

Mutation of the prototypic CCP family member, Nna1 (also known as CCP1), was found to underlie the phenotype of *Purkinje cell degeneration (pcd*) mice^11^. The *pcd* mice exhibit male infertility and degeneration of certain neurons, including cerebellar Purkinje cells, retinal photoreceptors, and olfactory bulb mitral cells^12^,^13^,^14^,^15^ (reviewed in ref. 16). Although Nna1, CCP4, and CCP6 have similar enzymatic properties towards tubulin, neither CCP4 nor CCP6 substitute for Nna1 in rescuing the *pcd* phenotype in mice^4^. Furthermore, the phenotypes associated with CCP4- and CCP6-deficiency also differ from that of *pcd* mice. CCP6-null mice exhibit no obvious neural degeneration or locomotor disorders, but rather have enlarged spleens and defective hemostasis with underdeveloped megakaryocytes and dysfunctional platelets^17^. In humans, *CCP4* mutations have been linked to dominant, late-onset Fuchs corneal dystrophy^18^. Recently, both CCP2- and CCP3-null mice have been generated, but exhibit no overt phenotypes^3^, further emphasizing that these apparently similar enzymes have distinct functions *in vivo*.

Unlike the other CCP family members that catalyze the hydrolysis of α-carboxyl–linked glutamate, CCP5 is the only CCP that cleaves the branching γ-carboxyl–linked glutamate^1^. Given this unique property, we anticipated that CCP5 would have indispensable physiological roles. *Caenorhabditis elegans* and *Drosophila melanogaster* have only two *CCP* genes, and one of the two belongs to the same phylogenic clade as mammalian *CCP5*^19^. In *C. elegans*, ccpp-6 is functionally more similar to CCP5 than to other CCPs and is involved in microtubule (MT) deglutamylation in sensory cilia^19^. In zebrafish, which express four *ccp* genes (i.e., *ccp1, ccp2, ccp5*, and *ccp6*), only *ccp5* deficiency leads to cilia MT hyperglutamylation and motility defects that induce a typical spectrum of ciliopathy phenotypes (e.g., axis curvature, pronephric cysts, and hydrocephalus)^20^. These findings suggest a specific function of CCP5 in ciliogenesis in lower species; however, the biological function of CCP5 in mammals is unknown.

There is a controversy as to whether CCP5 only metabolizes the branching glutamate^1^ or it can also catalyze removal of the α-carboxyl–linked glutamate^21^. In this study, we employed synthetic substrates to determine the substrate specificity of mammalian CCP5. We further assessed the ability of CCP5 to rescue Purkinje cell degeneration in *pcd* mice and investigated Purkinje cell survival and spermatogenesis in *Agbl5*-null mice as well as mice with compound deficiencies in *Agbl5* and *Nna1*.

## Results

### CCP5 specifically metabolizes γ-carboxyl-linked glutamate

**Figure 1:**
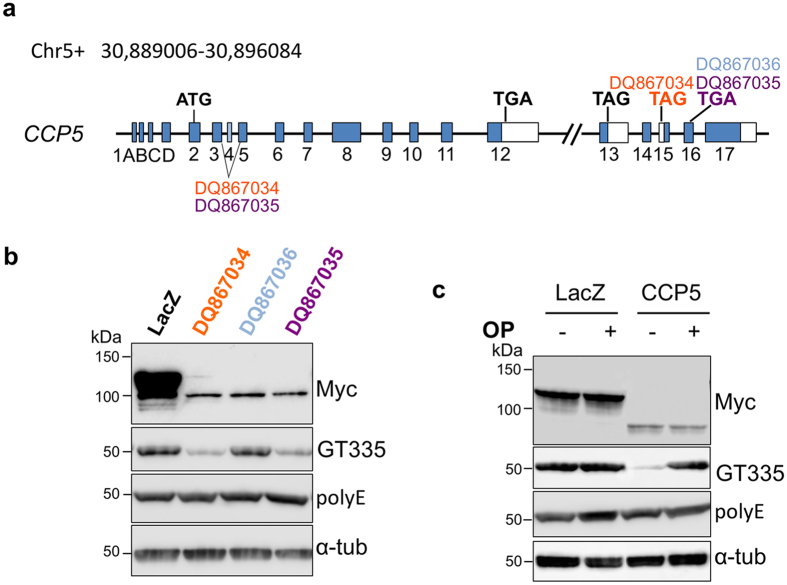
Enzymatic activities of CCP5 splicing variants. (**a**) Schematic representation of alternative splicing of *Agbl5* that generates three CCP5 variants. DQ867034 and DQ867035 do not contain exon 4 and use the stop codons in exon 15 and 16 respectively. DQ867036 is the only CCP5 variant that contains exon 4 and uses the stop codon in exon 16. (**b**) When porcine tubulin was incubated with the lysate of HEK293 cells transfected with CCP5 variants, DQ867034 and DQ867035, it exhibited reduced GT335 immunoreactivity compared to LacZ transfected cells, indicative of active enzyme. In contrast, lysates from DQ867036 transfected cells showed no change in tubulin GT355 immunoreactivity, indicative of an inactive enzyme under these conditions. None of the 3 CCP5 variants altered polyE immunoreactivity, indicating their inability to cleave α-carboxyl–linked glutamate. (**c**) The activity of CCP5 (DQ867034) on branching glutamate (GT335) is inhibited by addition of 5 mM 1,10-phenanthroline (OP).

Splicing variants of CCP5 exist in the mouse^22^ that mainly determine protein sequences at their C-termini. CCP5 splice variants DQ867034 and DQ867035 use stop codons in exon 15 and 16 respectively, leading to changes in the most C-terminal 20–30 amino acids. DQ867036 is the only isoform that contains exon 4 and uses a stop codon in exon 16, making it the longest CCP5 splice variant (Fig. 1a). As splicing might influence the enzymatic activity of CCP5, it was important to establish which CCP5 isoform to use for *in vitro* and *vivo* experiments.

To examine the enzymatic activities of the CCP5 isoforms (i.e. DQ867034, DQ867035 and DQ867036), we expressed them as N-terminal myc-tagged proteins in HEK293 cells. Lysates from the transfected cells were incubated with porcine brain tubulin and enzyme activity measured using immunoblotting with the GT335 antibody, which detects the branching glutamate^23^ (Fig. 1b). Specificity of reactions was confirmed using internal total tubulin controls and the generic metallocarboxypeptidase inhibitor, 1,10-phenanthroline (Fig. 1b,c, Supplementary Fig. S1). Compared to the myc-lacZ control, CCP5 variants DQ867034 and DQ867035 reduced GT335-immunoreactive signals (Fig. 1b) in a 1,10-phenanthroline-inhibitable fashion (Fig. 1c, Supplementary Fig. S1), indicating the removal of the branching-point glutamate and further suggesting the presence of variant exon 16 is not essential for CCP5 activity (Fig. 1b). DQ867036, which contains exon 4 and introduces 29–amino acids N-terminal to the carboxypeptidase domain, did not alter GT335-immunoreactivity, suggesting an isoform of CCP5 with undetectable activity, at least with tubulin as substrate at our conditions (Fig. 1b). In contrast to GT335, the immunoreactive signal with polyE antibody, which recognizes 3 or more consecutive glutamates at C-termini^1^,^24^,^25^, was not altered by any of these isoforms (Fig. 1b,c). This indicates that CCP5 specifically catalyzes removal of the branching glutamate, but does not metabolize runs of α-carboxyl-linked glutamate. Therefore, DQ867034 (referred to as CCP5 in subsequent text) was selected for further studies.

**Figure 2:**
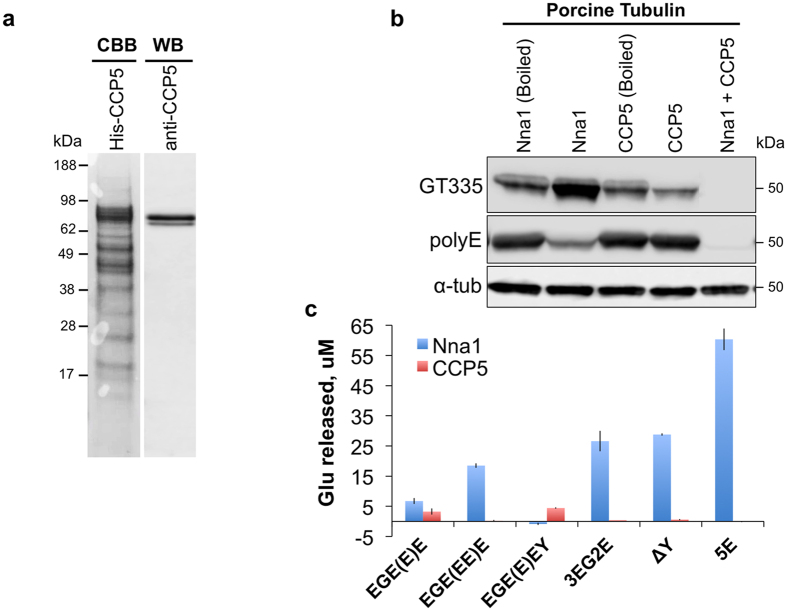
Purified recombinant CCP5 catalyzes the deglutamylation of porcine tubulin and synthetic substrates. (**a**) SDS-PAGE of purified recombinant CCP5 (DQ867034 splice variant) stained with Coomassie brilliant blue (CBB) (left lane). The major CBB band is immunoreactive with a CCP5 specific antibody (right lane). (**b**) Recombinant CCP5 and/or Nna1 were incubated with porcine tubulin and the deglutamylation activity was monitored by immunoblotting using GT335 and polyE antibodies. CCP5, but not the heat-denatured (Boiled) enzyme, reduced the GT335 signal without altering polyE immunoreactivity, indicative of specific removal of the branching glutamate of tubulin. Nna1 alone substantially reduced polyE immunoreactivity, though it increased the GT335 signal. Co-incubation of Nna1 and CCP5 completely abolished GT335 signal and further reduced polyE signal. (**c**) CCP5 is not active against three Nna1 synthetic substrates (Biotin-3EG2E, Biotin-ΔY, and Biotin-5E^4^), but it is active against a substrate with an exposed γ-carboxyl-linked glutamate (Biotin-EGE(E)E). When the γ-carboxyl-linked glutamate is in chain with another α-linked glutamate (Biotin-EGE(EE)E), CCP5 is no longer active. CCP5, but not Nna1, is active against Biotin-EGE(E)EY, where the only terminal glutamate is linked through a γ-carboxyl, further confirming their substrate specificities. Bars are mean ± SEM (error bars) of triplicate determinations.

To characterize the enzymatic properties of CCP5 a recombinant N-terminal histidine-tagged CCP5 was prepared^21^ (Fig. 2a). The major Coomassie Brilliant Blue band was of the correct predicted molecular mass on SDS-PAGE and was immunoreactive with a CCP5-specific antibody (Fig. 2a). When incubated with porcine tubulin, the CCP5-containing fraction reduced but did not eliminate the GT335 signal and its prior heat-denaturation abolished this activity (Fig. 2b, Supplementary Fig. S2). Nna1 is reported to catalyze the removal of α-carboxyl-linked glutamate but not the branching glutamate^1^,^19^. Indeed recombinant Nna1 decreased polyE signal and increased GT335 signal (Fig. 2b, Supplementary Fig. S2). Critically, when Nna1 and CCP5 were co-incubated with porcine tubulin, the GT335 signal was completely abolished (Fig. 2b, Supplementary Fig. S2). These results suggest that: (a) porcine tubulins harbor a range of polyglutamate chain sizes, including single residue chains, that are the substrate for CCP5; (b) longer chains are metabolized by Nna1 resulting in the accumulation of monoglutamate side chains; (c) CCP5 can only metabolize the branch point γ-linked glutamate after the α-linked glutamates are removed; (d) as there is residual GT335-positive tubulin after treatment with CCP5 the antibody must still bind longer polyglutamate chains but presumably with lower avidity.

We next quantified glutamate release from biotin-based synthetic substrates containing various lengths of α- and γ-linked glutamate chains that mimicked the amino acid sequence around the principle polyglutamylation site in tubulin^2^,^4^. As Nna1 can metabolize runs of α-linked polyglutamate residues in both the primary chain and side chains, we first assessed whether CCP5 could cleave α-linked glutamates using two synthetic peptides (Biotin-3EG2E and Biotin-ΔY) known to be excellent substrates for Nna1 and the related enzymes, CCP4 and CCP6^4^. Whereas Nna1 released glutamate from both substrates, CCP5 was inactive (Fig. 2c). As some CCPs prefer longer polyglutamate chains as substrates (Wu *et al*.^4^), we also tested CCP5 activity towards Biotin-5E. Whereas Nna1 released a high level of glutamate from Biotin-5E, CCP5 was inactive. These results suggest that CCP5 does not cleave α-linked glutamate residues. We next synthesized two new substrates, Biotin-EGE(E)E and Biotin-EGE(EE)E, that contain a γ-carboxyl-linked glutamate side chain at the −1 position relative to the C-terminus. Biotin-EGE(E)E had one α-carboxyl–linked glutamate and one γ-carboxyl–linked glutamate exposed at termini, whereas in Biotin-EGE(EE)E an additional α-linked glutamate was attached to the γ-carboxyl–linked branching glutamate. The CCP5 fraction released glutamate from Biotin-EGE(E)E but not Biotin-EGE(EE)E (Fig. 2c) confirming that it could metabolize a γ-linked glutamate and that the presence of an α-linked glutamate on the branch glutamate inhibited CCP5-mediated cleavage of the γ-linked residue. Nna1 released glutamate from both substrates, however, this is most likely cleavage of the C-terminal α-linked glutamate in Biotin-EGE(E)E and the same C-terminal glutamate plus the α-linked glutamate in the side chain of Biotin-EGE(EE)E. This conclusion was confirmed using another substrate Biotin-EGE(E)EY, in which the only glutamate exposed at the terminus is linked through a γ-carboxyl. CCP5, but not Nna1 released glutamate from this substrate (Fig. 2c). These results also confirmed that Nna1 does not catalyze removal of γ-carboxyl–linked glutamate.

### Targeted expression of CCP5 in Purkinje cells does not rescue Purkinje cell death in *pcd* mice

**Figure 3:**
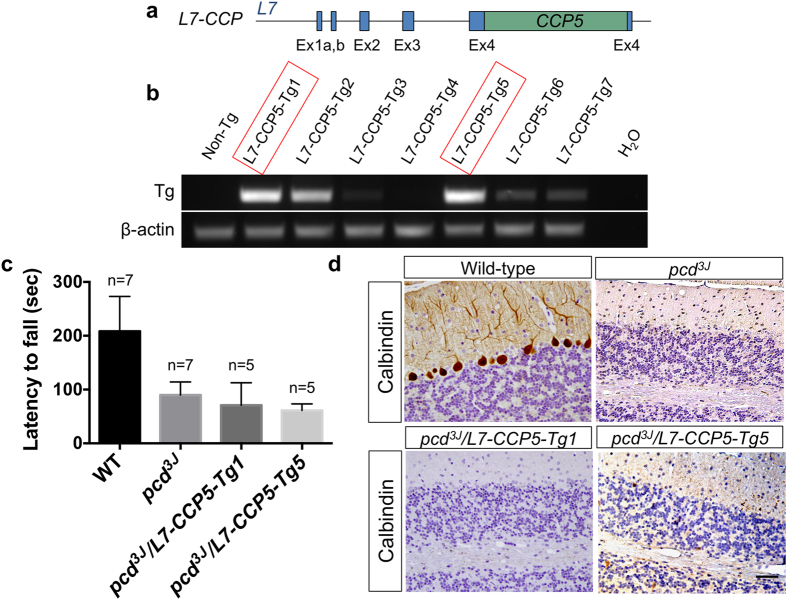
*CCP5* fails to rescue Purkinje cell death in *pcd* mice. (**a**) Schematic representation of *L7-CCP5* transgenes. *CCP5* cDNA (DQ867034) was inserted into a unique BamHI site in the fourth exon of the *L7* gene^36^. (**b**) RT-PCR to determine transgene expression in cerebellum of wild-type (non-Tg) and different *L7-CCP5* transgenic lines. The lines chosen for further investigation are boxed in red. (**c**) Accelerating rota-rod test of 2-month of age, gender balanced wild-type (WT) mice, *pcd*^*3J*−/−^ and *pcd*^*3J*−/−^ mice harboring *L7-CCP5* (lines Tg1 and Tg5) showed that only the wild-type (WT) groups differed significantly (*p* < *0.05*) from the groups of *pcd*^*3J*−/−^ or either line of *pcd*^*3J*−/−^ harboring the transgene (ANOVA). Therefore, CCP5 did not improve locomotor scores in *pcd*^*3J*−/−^ mice. The bars represent standard error of the means. (**d**) Calbindin D-28K immunohistochemistry and hematoxylin counterstaining of cerebellar sections from 2-month old wild-type (WT), *pcd*^*3J*−/−^, *pcd*^*3J*−/−^ harboring 2 independent alleles (Tg1 and Tg5) of *L7-CCP5* transgene. Note loss of calbindin-positive Purkinje cells in 2-month old *pcd*^*3J*−/−^ mice, that is rescued by an *L7-Nna1* transgene^26^, whereas neither line of *L7-CCP5* transgene prevents Purkinje cell death in *pcd*^*3J*−/−^ mice. Scale bar: 50 μm.

Loss of Nna1 function, as occurs in *pcd* mice, should result in a shift in polyglutamylation dynamics to favor longer chain lengths. Indeed, tubulin from the cerebella of *pcd* mice exhibited elevated levels of GT335-immunoreactivy that migrates more slowly than that of wild-type control on a SDS-PAGE (see below). As CCP5 reduces GT335-signal in *vitro*, it should reduce the pool of monoglutamylated protein, the substrate of further chain elongation, and thus reduce total protein polyglutamylation. Therefore, we tested whether introducing CCP5 into Purkinje cells would rescue their death in *pcd* mice. Previously, we demonstrated that targeted expression of Nna1, but not catalytic site mutants of the enzyme, in Purkinje cells using the cerebellar Purkinje cell–specific *L7/pcp2* promoter rescued the Purkinje cell degeneration and related locomotor deficits in *pcd* homozygous mice^2^,^26^. Using the same strategy, we constructed *L7-CCP5* (Fig. 3a) and generated multiple independent founder lines of transgenic mice (Fig. 3b). We selected the two lines with the highest mRNA expression levels of the fusion transgene (red-boxes, Fig. 3b) for investigation and crossed them into the homozygous *pcd*^*3J*^ background.

At 6 to 7 weeks of age, all *pcd*^*3J*^ homozygous mice and *pcd*^*3J*^ homozygous harboring *CCP5* transgenic alleles were ataxic, whereas those harboring a wild-type *Nna1* transgenic allele were indistinguishable from wild-type mice^26^. To quantify locomotor coordination, transgenic mice were tested on an accelerating rota-rod (Fig. 3c). The performance of *pcd*^*3J*^ mice was markedly impaired compared to that of wild-type mice, and transgenic expression of *CCP5* failed to improve those locomotor scores (Fig. 3c). Therefore, unlike Nna1, CCP5 failed to rescue this functional deficit in *pcd*^*3J*^ mice.

After the behavioral evaluation, we euthanized the mice and assessed cerebellar Purkinje cell survival via immunohistochemical analysis using anti-calbindin-D28K, a marker for Purkinje neurons^27^ (Fig. 3d). At 8 weeks of age, *pcd*^*3J*^ homozygous mice have lost almost all cerebellar Purkinje cells compared to wild-type mice, and neither lines of the *L7-CCP5* transgene spared the Purkinje cells death on a *pcd*^*3J*^ homozygous background (Fig. 3d). Thus, CCP5 cannot replace or compensate for Nna1’s loss of function in Purkinje cell survival.

### *Agbl5*-KO mice do not recapitulate the neurodegeneration phenotype of *pcd* mice

**Figure 4:**
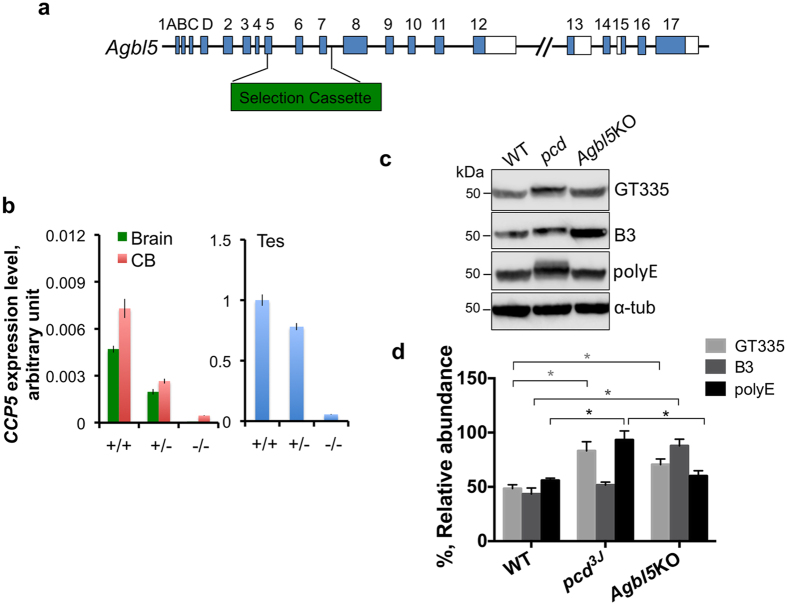
*Agbl5* deficiency increases protein glutamylation in cerebellum. (**a**) Schematic representation of *Agbl5*-KO allele. A region spanning exons 5, 6 and 7, which encodes ¾ of the entire carboxypeptidase domain of CCP5, is replaced by a selection cassette. (**b**) Quantitative real-time PCR using a probe spanning exon 11 and 12 shows that CCP5 RNA levels in the tissues examined are greatly reduced in heterozygous animals compared to that of wild-type littermates, and are barely detectable in the homozygous mutants. RNA levels were normalized to internal GAPDH levels and compared with the values of wild-type testis. Bars are mean ± SEM (error bars) of determinations of three animals. (**c**) Glutamylation or polyglutamylation levels in cerebellar lysates from wild-type (WT), *pcd*^*3J*−/−^ and *Agbl5*-KO mice are monitored by immunoblotting for GT335, B3, and polyE immunoreactivities, and (**d**) quantitatively analyzed using ImageStudio with normalization to α-tubulin levels. The bars represent the mean ± SEM of 3 animals of each genotype. GT335 immunoreactivity is significantly elevated in both *pcd* and *Agbl5-*KO mice compared to that of wild-type (*p* < 0.05), whereas B3 signal is only significantly increased in *Agbl5-*KO mice (*p* < 0.05) (Student’s *t* test). PolyE immunoreactivity is significantly increased in *pcd* cerebellum, but unaltered in *Agbl5*-KO cerebellum.

As CCP5 is the only enzyme known to catalyze the removal of the branching glutamate and given its ability to cooperate with Nna1 to completely remove polyglutamate chains in *vitro*, we asked whether its loss of function would cause neurobiological phenotypes either alone or in combination with partial or complete loss of Nna1 function. We obtained an allele containing the targeted deletion of exons 5, 6, and 7 in *Agbl5* (Fig. 4a), which leads to the loss of a large region of the carboxypeptidase catalytic domain in CCP5. Using a TaqMan® probe spanning exon 10–11 of *Agbl5*, we quantitatively assessed RNA levels of *Agbl5* in the cerebellum, brain, and testis (Fig. 4b). Compared to wild-type mice, *Agbl5* levels were reduced in *Agbl5*-heterozygous mice, and were almost undetectable in *Agbl5*-homozygous mutants (Fig. 4b).

The glutamylation status of tubulin in cerebellum of *Agbl5* mutant mice was analyzed using the GT335 antibody (which recognizes the branching glutamate^23^), B3 antibody (which recognizes a side chain with 2 or more glutamate residues^28^,^29^), and polyE antibody (which recognizes 3 or more glutamate residues^1^,^24^,^25^). In *Agbl5*-KO mice, both GT335 and B3 signal was enhanced in cerebellum (Fig. 4c,d), but polyE signal was not altered. This indicates that loss of *Agbl5* results in an increased pool of monoglutamylated tubulin that can be extended further by one more glutamate, but longer chains are presumably degraded by Nna1. This hypothesis is supported by findings in *pcd* cerebellum, where the polyE signal is significantly enhanced, indicating an increase in longer chain (≥3E) populations (Fig. 4c,d and ref. 1). Also, unlike in *pcd* mice (Fig. 4c), in *Agbl5*-KO cerebellum we did not observe the characteristic retarded shift in migration of glutamylated tubulin on SDS-PAGE (Fig. 4c)^2^.

**Figure 5:**
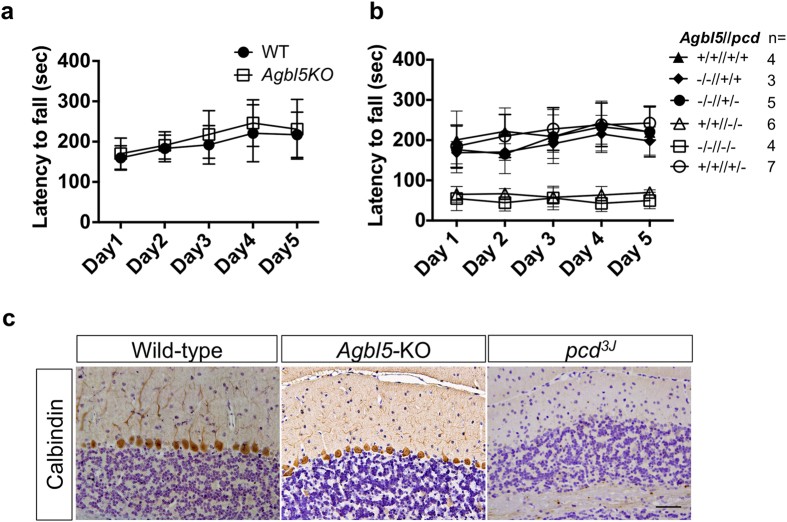
*Agbl5* deficit does not recapitulate the neurodegeneration phenotype of *pcd* mice. (**a**) 2-month-old gender-balanced littermates of wild-type (WT, n = 4) and *Agbl5*-KO (n = 9) were tested on an accelerating rota-rod for five consecutive days. The latency to fall in seconds for all animals of each group was recorded and presented as mean ± SEM. One-way ANOVA showed that the wild-type mice are not significantly different (*p* > *0.05*) from the *Agbl5-*KO. Therefore, *Agbl*5 deficit does not cause the locomotor dysfunction seen in *pcd*^*3J*−/−^ mice. (**b**) Rota-rod test to determine whether deletion of *Agbl5* exacerbates the locomotor phenotype in *pcd*^*3J*−/−^ mice or elicits a synthetic locomotor deficit in *pcd-*heterozygous mice. 2-month old gender-balanced littermates of each genotype (n = 3–7) were tested on a rota-rod as described. One-way ANOVA showed *Agbl5-*KO/*pcd* double mutants (−/−//−/−) are indistinguishable from *pcd* (+/+//−/−) mice. *Agbl5*-KO mice on a *pcd* heterozygous background (−/−//+/−) are not ataxic and have a locomotor performance comparable with that of wild-type (+/+//+/+), *Agbl5*-KO (−/−//+/+) and *pcd* heterozygous (+/+//+/−) mice. (**c**) Calbindin D-28K immunohistochemistry and hematoxylin counterstaining of cerebellar sections from 2-month old wild-type, *Agbl5-*KO, and *pcd*^*3J*−/−^ mice. In contrast to their loss in *pcd*^*3J*−/−^ mice, calbindin-positive Purkinje neurons are preserved in *Agbl5*-KO similar to those in wild-type animals. Scale bar: 50 μm.

Locomotor coordination and locomotor learning of *Agbl5*-KO mice was assessed using an accelerating rota-rod for 5 consecutive days. *pcd*^*3J*^ mice have markedly impaired locomotor performance (ref. 26 and Fig. 5b). However, the locomotor scores of *Agbl5-KO* mice were not significantly different from those of wild-type littermates (Fig. 5a,b). Calbindin immunohistochemistry was also employed to assess whether there might be subtle loss of Purkinje cells in *Agbl5*-null mice. Analysis of the cerebella of 8-week-old wild-type, *Agbl5*-KO, and *pcd*^*3J*^ mice revealed that calbindin-D28K–positive neurons were nearly absent in the *pcd*^*3J*^ mice, but the number of calbindin-D28K–positive neurons in *Agbl5-KO* mice was comparable to that in wild-type mice (Fig. 5c). Therefore, CCP5 is not essential for cerebellar Purkinje cell survival.

We next questioned whether an *Nna1* and *Agbl5* double-mutant exhibited a more severe phenotype than loss of *Nna1* alone or if deletion of *Agbl5* would elicit a synthetic phenotype in *pcd-*heterozygous mice. The locomotor performance of *Agbl5-*KO/*pcd* double-mutants did not differ from that of *pcd* mice (Fig. 5b). In *pcd* mice, olfactory bulb mitral cells degenerate with a slower time course than Purkinje cells^14^. This afforded the opportunity to assess whether concomitant loss of *Nna1* and *Agbl5* enhanced or retarded degeneration of these neurons. In 6-month old *pcd* or *Agbl5*-KO/*pcd* mice, immunohistochemistry with anti-Tbr2 (a marker for olfactory bulb mitral cells^30^) showed that there was no obvious difference in the level of degeneration of mitral cells (Supplementary Fig. S3). Furthermore, knocking out *Agbl5* did not cause any ataxic phenotypes in *pcd*-heterozygous animals (Fig. 5b).

### *CCP5* expression in testis

**Figure 6:**
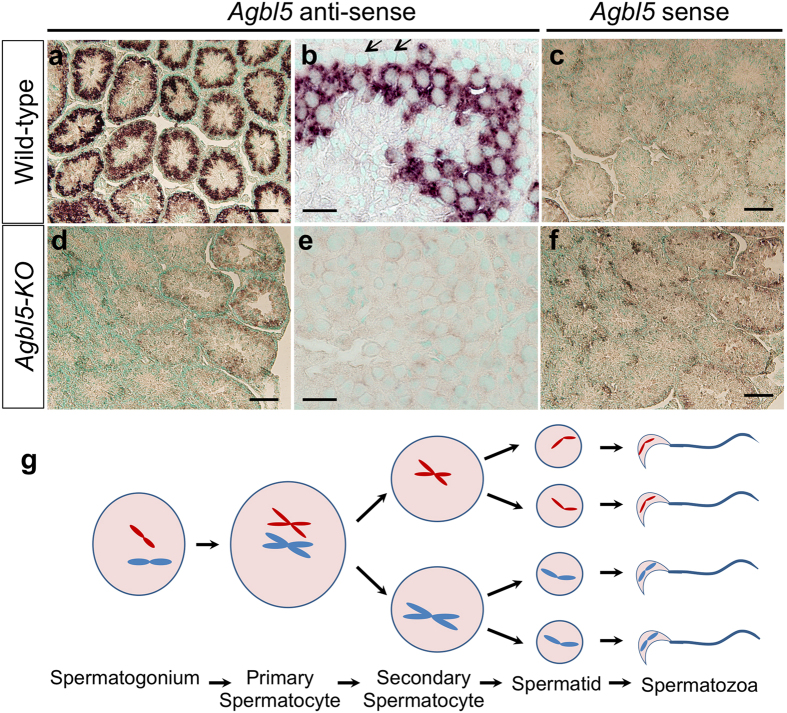
*In situ* hybridization of CCP5 on testis. Digoxin-labeled RNA probes that span exon 2–7 of CCP5 cDNA are used to determine CCP5 expression on testis sections of 3-month old wild-type (**a**–**c**) and *Agbl5*-KO (**d**–**f**) mice. The antisense probes reveal CCP5 is prominently expressed in developing germ cells from spermatocytes onward but is undetectable in spermatogonia (arrows) in wild-type mice (**a**,**b**). The same probe does not hybridize on *Agbl5*-KO testis (**d**,**e**). The digoxin-labeled sense RNA probe hybridizes neither on wild-type nor *Agbl5*-KO testis (**c**,**f**). Sale bars: 100 μm in (**a**,**c**,**d**,**f**); 20 μm in (**b** and **e**). (**g**) Schematic representation of spermatogenesis. Based upon their location in the testis (Supplementary Fig. S4), the cells expressing *CCP5* are likely primary and secondary spermatocytes.

As *CCP5* RNA levels are about 100-time higher in testis than cerebellum or brain (Fig. 4b) we performed *in situ* hybridization using a digoxigenin-labeled RNA probe to identify the cellular sources of CCP5 in testis. A schematic representation of spermatogenesis is shown in Fig. 6g. In wild-type testis, *CCP5* was highly expressed in developing germ cells (Fig. 6a,b). Expression was predominantly in spermatocytes and their descendants, but was absent in spermatogonia (arrows, Fig. 6b). Based on their location in testis (Supplementary Fig. S4), the *CCP5*-positive cells are probably primary and secondary spermatocytes, suggesting a role for CCP5 in spermatogenesis. Using the same antisense probe, no signal was detected in *Agbl5-*KO testis (Fig. 6d,e); digoxigenin-labeled sense probe failed to produce signal in both wild-type and *Agbl5-*KO testis (Fig. 6c,f).

### *Agbl5* deficiency results in impaired spermatogenesis

**Figure 7:**
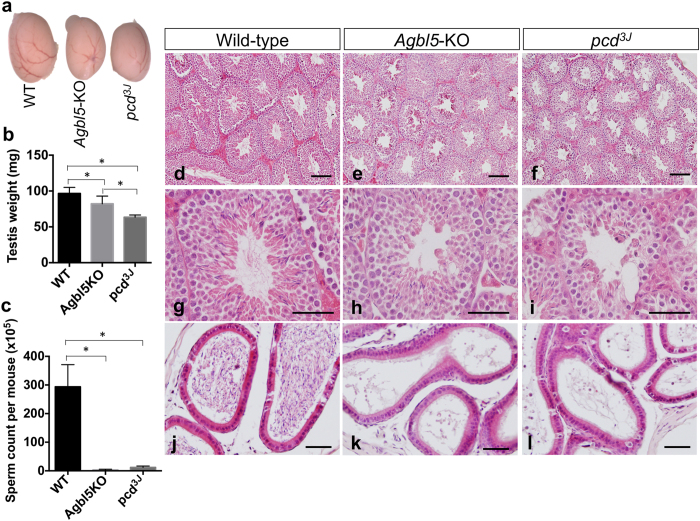
Defective spermatogenesis in *Agbl5*-KO mice. (**a**) Testes of 3-month old *Agbl5*-KO and *pcd*^*3J*−/−^ mice are smaller than those of age-matched wild-type mice. (**b**) Comparison of the weight of testes from 3-month old wild-type (WT, n = 8), *Agbl5*-KO (n = 8), and *pcd*^*3J*^ (n = 4) mice. Although testes from both *Agbl5-*KO and *pcd*^*3J*^ mice weigh significantly less (*p* < 0.05) than those of wild-type mice, *Agbl5*-KO testes weigh more than those from *pcd*^*3J*^ mice (*p* < 0.05) (Student’s *t* test). (**c**) The sperm counts from epididymis and vas deferens of *Agbl5*-KO and *pcd*^*3J*^ are significantly lower than those of wild-type mice (*p* < 0.05) (Student’s *t* test). (**d**–**l**) Hematoxylin-Eosin staining on sections of testis (**d**–**i**) and epididymis (**j**–**l**) from wild-type (**d**,**g**,**j**), *Agbl5*-KO (**e**,**h**,**k**) or *pcd*^*3J*^ (**f**,**i**,**l**) mice. Markedly fewer mature sperm are observed in *Agbl5*-KO (**e**,**h**) and *pcd*^*3J*^ testes (**f**,**i**) compared to wild-type (**d**,**g**) mice. The thickness of the testicular epithelium is reduced in *pcd*^*3J*^ mice (**f**,**i**) but not in *Agbl5*-KO mice (**e**,**h**). The epididymis of wild-type mice is fully filled with mature sperm (**j**). In contrast, only a few mature sperm reside in the epididymis of *pcd*^*3J*^ (**l**) mice and even less are observed in *Agbl5*-KO mice (**k**). Scale bars: 100 μm in (**d**–**f**); 50 μm in (**g**–**l**).

Progeny testing showed that male but not female *Agbl5*-KO mice were infertile. The testes of *Agbl5-*KO mice were smaller than wild-type animals (Fig. 7a,b) and the sperm count from epididymis and vas deferens was about 100-time lower (Fig. 7c). Although *pcd* mouse testis was even smaller than *Agbl5*-null mice (Fig. 7a,b) their sperm count was not as low albeit still much reduced from wild type levels (Fig. 7c). Hematoxylin-eosin staining revealed that *Agbl5*-KO testes showed less severe overall morphologic changes than *pcd* testes, where the thickness of the germinal epithelium was dramatically reduced, and substantial cell loss was evident, mainly from the spermatocyte stage onward (Fig. 7d–f)^31^. In *Agbl5-*KO mice, the main alteration of the germinal epithelium was the greatly reduced number of spermatozoa (Fig. 7h). In the epididymis of wild-type mice, numerous mature sperm were present (Fig. 7j). In contrast, only a few mature sperm were identified in *Agbl5-*KO and *pcd* epididymis sections (Fig. 7k,l). These results suggest that CCP5 loss affects spermatogenesis at a late stage.

**Figure 8:**
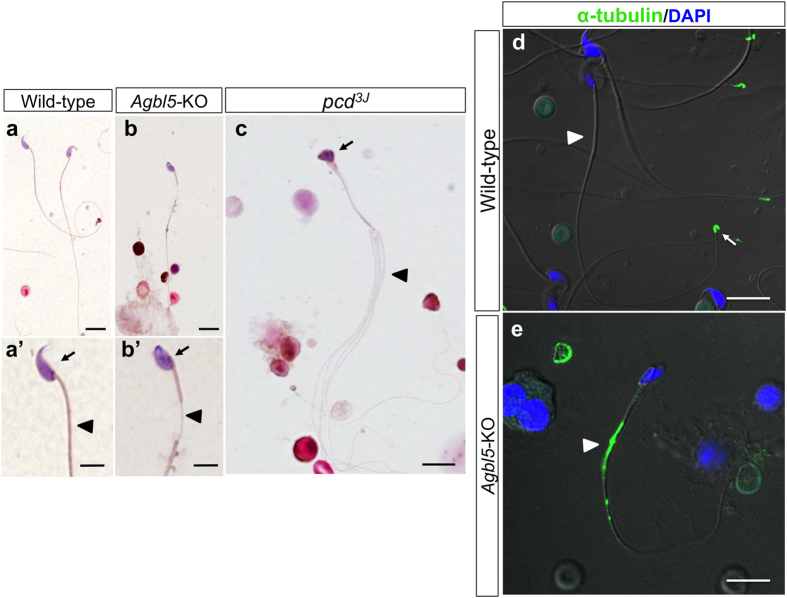
Abnormal mature sperm in *Agbl5*-KO mice. (**a**–**c**) Hematoxylin-Eosin staining on sperm from epididymis and vas deferens of wild-type (**a**,**a’**), *Agbl5*-KO (**b,b’**), and *pcd*^*3J*^ (**c**) mice. The heads of sperm from *Agbl5*-KO mice are abnormally bent (**b’**, arrow) and segments of their tails are not ensheathed (**b’**, arrow head). Sperm from *pcd*^*3J*^ mice also have aberrant heads (**c**, arrow) and they frequently have multiple tails (**c**). (**d**–**e**) Mature sperm from epididymis and vas deferens of wild-type (**d**) and *Agbl5*-KO (**e**) mice are immunostained with α-tubulin antibody (ascites fluid B-5–12, green) and the nuclei visualized with DAPI (blue) staining. While α-tubulin antibody only stains the end piece of wild-type sperm (d, arrow), a large region in the middle of the tail of *Agbl5*-KO sperm is immunoreactive to the same antibody, indicating an unwrapped flagellum (**e**, arrow head). Scale bars: 10 μm in (**a**,**b**,**c**,**d**, and **e**); 5 μm in (**a’** and **b’**).

Compared to sperm released from the epididymis and vas deferens of wild-type mice, those from *Agbl5-*KO mice had an abnormally shaped head (Fig. 8b,b’, arrow,) and a tail flagellum that was not fully wrapped by the sheath (arrow head, Fig. 8Ab’). Consistent with earlier studies^14^,^31^ the sperm from *pcd* mice also had an aberrant head, and often had multiple tails (arrow head, Fig. 8c). Thus, as in the whole testis, there were both common and distinct aspects of sperm phenotypes in *pcd* and *Agbl5*-null mice. When wild-type sperm were stained with α-tubulin antibody, the immunoreactive signal was detectable only at the end piece (arrow, Fig. 8d). However, we often observed α-tubulin–immunoreactivity in the middle-portion of the main piece in the tail of *Agbl5-*KO sperm, a location associated with incomplete ensheathment (arrow head, Fig. 8e). These data suggest that CCP5 is required for proper tail formation, presumptively through its ability to regulate its state of polyglutamylation.

### Ectopic tubulin polyglutamylation in developing sperm cells of *Agbl5*-KO mice

**Figure 9:**
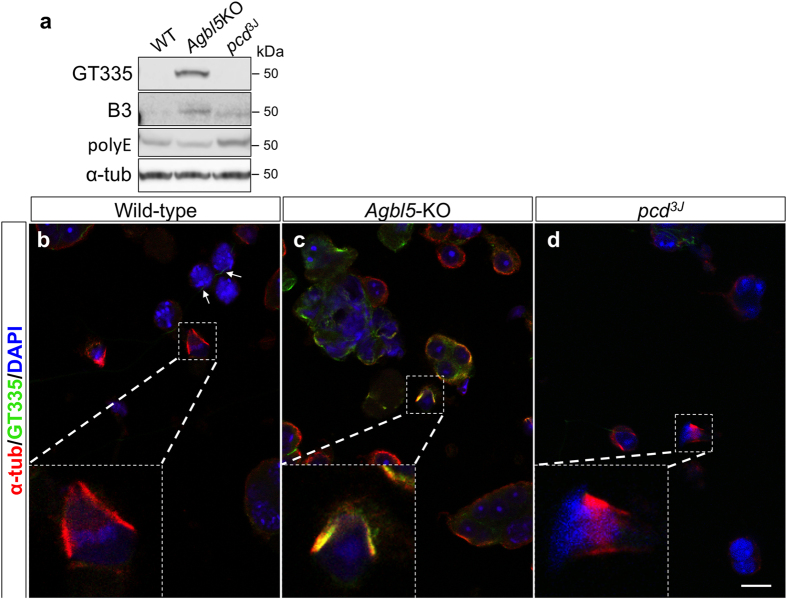
Ectopic tubulin polyglutamylation in developing sperm cells of *Agbl5*-KO mice. (**a**) Glutamylation and polyglutamylation levels in lysates of testis from 3-month old wild-type (WT), *Agbl5*-KO and *pcd*^*3J*^ mice are monitored by western-blot for GT335, B3, and polyE immunoreactivities. Both GT335 and B3 signals are prominently increased in *Agbl5*-KO testis compared to that of wild-type, but polyE immunoreactivity remains unchanged. B3 and polyE signals are marginally increased in *pcd*^*3J*^ testis. (**b**–**d**) Dissociated developing sperm from wild-type (**b**), *Agbl5*-KO (**c**) or *pcd*^*3J*^ (**d**) mice are co-immunostained for α-tubulin (EP1332Y, red) and glutamylation (GT335, green) and nuclei are visualized with DAPI staining (blue). In wild-type mice, GT335 immunoreactivity is mainly detectable in the flagellum of the developing sperm (**b**, arrows), whereas in *Agbl5*-KO mice profound GT335 signal is detected in the cell body of developing sperm (**c**). Notably, GT335 signal is undetectable in the manchette of wild-type spermatids (**a**, insert, and ref. 32) but is prominently expressed in manchette of *Agbl5*-KO (**c**, insert) mice. Similar to wild-type mice, in *pcd* mice GT335 immunoreactivity is largely absent in the manchette (**d**, insert). Sale bar: 10 μm.

We examined tubulin glutamylation status in the testis of *Agbl5-*KO mice using GT335, B3 and polyE antibodies. The basal level of tubulin polyglutamylation in wild-type testis is low with minimal B3 signal and no signal with GT335 (Fig. 9a). In *pcd* mice, there is also no GT335 signal, although there is detectable B3-immunoreactivity and slightly increased polyE signal (Fig. 9a and ref. 1), suggesting that loss of Nna1 causes longer glutamate chains, but does not expand the pool of total glutamylated tubulin. In contrast, in *Agbl5*-KO mice there is a prominent signal with GT335 and an increased signal with B3 (Fig. 9a), suggesting that loss of CCP5 expands the pool of monoglutamylated tubulin and provides more substrate for further chain elongation. However, as in cerebellum (Fig. 4c), *Agbl5* deficiency did not increase polyE immunoreactivity in testis, suggesting a limitation of net elongation. Furthermore, the slowed migration of the B3 bands in *Agbl5*-null testis is indicative of increased chain length (Fig. 9a). As loss of CCP5 leads to a greater pool of polyglutamylated tubulin compared to Nna1 loss, CCP5 is apparently the major contributor to the catabolism of polyglutamate chains on tubulin and CCP5 and Nna1 may have distinct substrates of different abundances in testis or developing male germ cells.

To understand *Agbl5* functions in spermatogenesis, dissociated developing germ cells from adult wild-type, *Agbl5*-KO and *pcd* testis were co-immunostained with α-tubulin and GT335 antibodies. In wild-type mice, GT335 immunoreactivity is confined to the tail of spermatids (arrows, Fig. 9b) and is largely absent in the cytoplasm of developing sperm cells (Fig. 9b). In contrast, strong GT335 signals were detected in the cytoplasm of the developing sperm cells of *Agbl5*-KO mice (Fig. 9c). The spermatid manchette, which is normally negative for GT335 signal (Fig. 9b and insertion and ref. 32), is prominently labeled in *Agbl5*-KO mice (Fig. 9c and insertion). In contrast, the GT335 signal in *pcd* spermatid manchettes is similar to that of wild-type (Fig. 9d and insertion). Furthermore, this is not the result of increased CCP5 expression in *pcd* mice (Supplementary Fig. S5), emphasizing that these two CCP family members function at different stages or processes of spermatogenesis.

Collectively, these data indicate that CCP5 plays an important role in spermatogenesis through mechanisms involved in establishing structures or pathways for shaping the sperm head and forming the tail. Although Nna1 deficiency also causes abnormal spermatogenesis, the two CCP family members appear to play different roles in this process.

## Discussion

The finding that loss of function mutations in Nna1/CCP1 underpinned the phenotype of *pcd* mice, first implicated disrupted polyglutamylation in causing defects in brain, testis and retina^1^,^11^. Although CCP5 activity catalyzing α-carboxyl linked glutamate was detected in tubulin^21^, using synthetic model substrates, we can only detect its activity on the γ-carboxyl linkage (Fig. 2c). As CCP5 is thought to be the only enzyme capable of removing the branch point glutamate, it was anticipated that its loss would cause a global disruption of protein polyglutamylation by increasing the pool of mono-glutamylated substrates, leading directly to aberrant phenotypes and potentially exacerbating the phenotype of complete or partial deficiency in other CCPs such as Nna1. However, loss of CCP5 does not mimic the neurodegeneration seen in *pcd* mice, nor does targeted over-expression of CCP5 rescue Purkinje cell degeneration in *pcd* mice (Figs 3, 4 and 5). In addition, heterozygous *pcd* mice that are also CCP5-null do not exhibit any overt nervous system phenotype and mice completely lacking both Nna1 and CCP5 are indistinguishable from homozygous *pcd* mice (Fig. 5). Thus, despite the fact that CCP5-deficiency leads to enhanced levels of polyglutamylation in mice and loss of function of its ortholog in *C.elegans* and zebrafish cause a spectrum of severe ciliopathy-related phenotypes^20^,^33^, the phenotype in CCP5-null mice is restricted to testis.

The present data add to the growing body of literature indicating that whereas all CCP family members catalyze the removal of glutamate from polyglutamate chains, their *in vivo* biological properties are distinguishable. All six CCP family members are abundantly expressed in the mouse testis^3^,^22^ yet only Nna1- and CCP5-deficiencies cause male infertility (refs 3, 11, 14 and 17 and this study). Knocking out CCP2, CCP3, or both does not overtly affect male fertility, despite modestly increased tubulin glutamylation in testis^3^. Similarly, mice lacking CCP6 are also reported to be fertile^17^. In contrast, male *pcd* mice are infertile and display reduced sperm count, but their testicular phenotype is distinguishable from that of *Agbl5-*KO mice (ref. 31 and Fig. 7). The *pcd* testis displays obvious defects in testicular structure accompanied by cell loss in the seminiferous tubules^31^ whereas the *Agbl5-*KO testis has no obvious cell loss. The major abnormalities of *Agbl5-*KO testes were a reduced number of spermatozoa, leading to few mature sperm in the epididymis and vas deferens. Some sperm released from *pcd* mice appeared to have a multinucleated head that was often attached to multiple tails. This phenotype is consistent with the proposal in Kim *et al*.^31^ that Nna1 plays a role in the meiosis stages of spermatogenesis. In contrast, this phenotype was not observed in sperm from *Agbl5*-KO mice. Rather we found that *Agbl5*-KO sperm had a bent head, and the flagellum of the tail was not completely covered by the sheath. These results suggest that CCP5 mainly functions during the late stages of spermatogenesis.

Male gamete development relies heavily on the coordinated assembly and rapid remodeling of microtubule structures, such as the spindle of mitotic and meiotic processes, the flagellum required for sperm motility, and the manchette that determines sperm head shape and contributes to the tail structure^34^. In wild-type mice, GT335 immunostaining, a surrogate marker for monoglutamylated tubulin is largely confined to the tails of spermatids and is not detected in the cytoplasm of developing sperm (Fig. 9b and ref. 32). In contrast, there is prominent GT335 staining in the cytoplasm and manchette of spermatids in *Agbl5*-null mice (Fig. 9c). A similar phenotype is observed in *olt* mice that also exhibit a failure in tail formation^35^. However, it is unclear whether the genes disrupted in *olt* encode proteins involved in polyglutamylation either as substrates or enzymatic regulators. Nevertheless, the aberrant spermatogenesis in *pcd* and *Agbl5*-KO mice suggests that (de) glutamylation of microtubules may affect multiple stages of spermatogenesis, and each step may require different enzymes (or combinations thereof) to maintain a balanced glutamylation status to facilitate normal sperm development.

The present findings not only add to the growing body of evidence that not all CCPs have equivalent functions *in vivo*, but they also raise the broader question of how the dynamics of protein glutamylation is regulated. The general view is one in which initiator TTLLs add the branch point glutamates and elongation TTLLs extend the glutamate chain. CCPs on the other hand are responsible for degrading the chain and CCP5 is solely responsible for metabolizing the γ-linked branching glutamate. Kinetic studies using tubulin and synthetic model substrates have shown that CCPs have distinguishable preferences for glutamate chain length and amino acid composition flanking the polyglutamate chain^4^. Moreover, multiple splicing variants exist for a number of CCPs, including CCP5, and at least one of the CCP5 variants appears to have distinct properties (Fig. 1). The fact that loss of CCP5 does not have broad ranging phenotypes and does not produce a synthetic phenotype in *pcd* heterozygous mice (Figs 4 and 5), may point to additional levels of regulation of polyglutamylation. Although CCP5 is the only enzyme identified to date that metalizes the branching glutamate, it is conceivable that another enzyme exists that has redundant function with CCP, thereby accounting for a milder phenotype in CCP5 null mice compared to *pcd* mice. A second possibility is that of feedback regulation in the expression and/or activity of TTLLs or CCPs in response to changes in glutamate chain length. A third possibility is that turnover of critical polyglutamylated proteins can be rate limiting. The *Agbl5*-null mouse provides a platform with which to begin to address these possibilities.

## Methods

### Animals

*pcd*^*3J*+/−^ and FVB/NJ mice were purchased from the Jackson Laboratory (Bar Harbor, ME, USA). The *Agbl5-*KO mice were obtained from Taconic Biosciences, Inc. (Catalog number: TF2926). 4 FVB/NJ females were used for pronuclear injection to generate *L7-CCP5* transgenic mice. Five *L7-CCP5* transgenic lines were generated, of which two independent lines were characterized. Subsequently, each transgenic line was crossed repeatedly with *pcd*^*3J*^ heterozygous mice to produce *pcd*^*3J*−/−^/*Tg* mice and all intermediate genotypes and 5 to 10 animals per genotype of interest were used in this study. *Agbl5* heterozygotes (*Agbl5*^+/−^) were intercrossed or crossed with *pcd* heterozygotes (*pcd3J*^+/−^). Subsequently, all strains of mice were multiply intercrossed to get the desired genotypes. Depending on the assay, between 3 and 21 animals per genotype were used. Animals were maintained on a 12-h light: 12-h dark cycle with free access to food and water. All studies were approved by the St. Jude Children’s Research Hospital (SJCRH) Animal Care and Use Committee (ACUC) and complied with the standards set forth in National Institute of Health Guide for the Care and Use of Laboratory Animals (NIH Publication No. 80–23, revised 1996).

### Generation of CCP5 transgenic mice

Blunt ended PCR products of the ORF of mouse *CCP5* (DQ867034) were inserted into the unique BamHI site of the fourth exon of the *L7* gene in a pGEM-3 vector^36^. The *L7-CCP5* transgenes were released from the vector, purified, sequenced and subjected to pronuclear injection as described previously^2^,^26^,^36^. *L7-CCP5* transgene status was genotyped in offspring using primers 5′-GCCAACAAAACTCTCCACAGATGAAGAAC-3′ and 5′-TTATTGTTTTCAGGGGCCAGTGGG-3′. Genotyping of the wild-type and *pcd*^*3J*^ alleles of *Nna1* was performed as described^11^,^37^.

### Generation *Agbl5*-KO and *Agbl5*-KO/*pcd* double mutants

*Agbl5*^+/−^ mice were inbred to produce *Agbl5*^−/−^ and wild-type litter mates, or were bred with *pcd*^*3J*+/−^ mice to produce *Agbl5*^−/−^/*pcd*^*3J*−/−^ mice and all intermediate genotypes. Genotyping of wild-type and *Agbl5*-KO alleles was performed using primer pairs of 5′-ACATACCCTTAGCCTCACCAGTT-3′ and 5′-TTCATGTTCTTCCCACTTACTTACC-3′ and 5′-ACATACCCTTAGCCTCACCAGTT-3′ and 5′-CCCTAGGAATGCTCGTCAAGA-3′ respectively.

### RT-PCR analysis of transgene expression

Total RNA was extracted from mouse cerebellum and first strand cDNA was generated as described previously^4^. The levels of *L7-CCP5* chimeric mRNAs in transgenic mice were evaluated by RT-PCR with primers 5′-GGCTTCTTCAACCTGCTGAC-3′ and 5′-ACCATGACCTGTTGCCATTT-3′. *β-actin* was amplified as a loading control using primers described previously^4^.

### qRT-PCR analysis of CCP5 expression

Total RNA was extracted from mouse cerebellum, the rest of brain, and testis with Trizol reagent (Invitorgene) according to the manufacturer’s protocol followed by first strand cDNA synthesis using a High-Capacity cDNA Reverse Transcription Kit (Applied Biosystems, Foster City, CA, USA). CCP5 was amplified with a TaqMan® probe (Mm01220582_g1, Applied Biosystems). The RNA levels were quantified using the standard curve method and normalized to GAPDH levels (TaqMan® probe: Mm99999915_g1) of individual samples.

### Histology and immunohistochemistry

The procedures for histological analyses of cerebella were as described^26^,^38^. A rabbit anti-calbindin D-28K antibody (Chemicon, Temecula, CA, USA) and a rabbit anti-Tbr2 antibody (Abcam, Cambridge, MA, USA) were used at the dilution of 1:500 to visualize Purkinje cells and olfactory bulb mitral cells respectively, and immune complexes were revealed using a peroxidase-conjugated anti-rabbit kit and diaminobenzidine tetrahydrochloride (DAB) substrate (Vector Labs, Burlingame, CA, USA). After immunostaining, sections were counterstained with hematoxylin (Sigma-Aldrich, St Louis, MO, USA).

### *In situ* hybridization

A region that spans exon 2 to exon 7 of CCP5 cDNA was amplified using primers 5′-CCATTTAGGTGACACTATAGGCCTGACTGTGCTGAAACGGAATA-3′ and 5′-GGTAATACGACTCACTATAGGGGAGCTTCATTGTGGAGATTATTGGC-3′ (the underlined are SP6 and T7 promoters sequences, respectively). The resulting amplicon was used as the template to synthesize the digoxin labeled sense and anti-sense RNA probes with SP6 and T7 RNA polymerases, respectively. The *in situ* hybridization was performed on 5 μm paraffin sections according the procedure described previously^39^ followed by counterstaining with 0.1% methyl green.

### Sperm count, morphology, and testicular germ cell dissociation

Sperm from epididymis and vas deferens were counted using the protocol described previously^40^ with minor modifications by including sperm from vas deferens in counting. The sperm were then subjected to hematoxylin-eosin staining for morphological assessment.

Mouse testicular germ cells were isolated for immunofluorescence according to the protocol described by Fouquet *et al*.^32^. Briefly, the decapsulated testes were minced with razor blades in PBS-EDTA at room temperature followed by 10 min agitation. Cells were recovered by filtration through a 100 μm nylon strainer and then centrifuged at 600 × g for 5 min.

### Immunofluorescence

Isolated testicular germ cells or spermatozoa released from the epididymis and vas deferens were fixed in PBS containing 4% paraformaldehyde for 30 min followed by three washes with PBS. Cells were permeabilized in cold acetone (−20 °C) for 10 min and washed with PBS (3 times, 5 min each time). The cells were then blocked with 0.2% BSA in PBS for 30 min followed by 2 h incubation with primary antibodies (EP1332Y 1:500; GT335, 1:1000; ascites fluid B-5-12 1:1000) in the same buffer. After 3 washes with PBS, cells were incubated with Alexa Fluor® 594 donkey anti-rabbit IgG (1:500) and/or Alexa Fluor® 488 goat anti-mouse IgG (1:500) for 1 h. Cells were incubated with PBS containing DAPI for 5 min followed by 3 washes with PBS. The cells were mounted with Prolong® Gold antifade reagent (Life Technologies, Eugene, OR, USA). Images were taken with a Zeiss LSM 710 NLO Confocal Microscope (Zeiss, USA).

### Preparation of recombinant mouse CCP5

A recombinant mouse CCP5 (DQ867034) construct containing a 6-histidine tag at the N-terminus was introduced into a Baculovirus vector (pFastBac-HT-B) and expressed in insect cells for large-scale protein production. The recombinant protein was purified using a method described previously^21^. Protein purity was monitored by Coomassie staining and immunoblotting.

### Protein electrophoresis and immunoblotting

Proteins were separated using a Criterion^TM^ XT precast gel (4–12% Bis-Tris, (Biorad, Hercules, CA, USA)). After electrophoresis, proteins were transferred onto a nitrocellulose membrane using the Criterion^TM^ Blotter (Biorad, Hercules, CA, USA). Membranes were incubated with rabbit anti-CCP5 (1:1000, Ab118621, Abcam, Cambridge, MA, USA), mouse anti-polyglutamate (B3, 1:2000, Sigma-Aldrich), mouse anti-glutamate (GT335, 1:4000, Adipogen, San Diego, CA, USA), rabbit anti-long-chain polyglutamate (polyE, 1:4,000, Adipogen), mouse anti-myc (1:1200, Clontech, Mountain View, CA, USA) or rabbit anti-α-tubulin (EP1332Y, 1:3000, Abcam) antibodies. Immunoreactivity of proteins was visualized with Supersignal® West Pico Chemiluminescence Substrate (Thermo, Rockford, IL, USA) following incubation with HRP-labeled sheep anti-mouse (1:2000, GE Healthcare Sciences, Pittsburgh, USA) or donkey anti-rabbit IgG (1:10,000, GE Healthcare Sciences) antisera.

### Assay of CCP5 carboxypeptidase activity

All synthetic substrates tested were synthesized in the Hartwell Center for Bioinformatics and Biotechnology at SJCRH. The activity of recombinant CCP5 on these synthetic substrates was determined by evaluating amino acids released according to methods described previously^4^,^41^.

### Tubulin glutamylase assay

To determine CCP activity towards polyglutamated tubulin, a 20 μl reaction mixture containing 1 μg of respective purified CCP5 and/or Nna1 and 2 μg porcine tubulin (Cytoskeleton Inc., Denver, USA) in PBS was incubated at 37 °C for 1 h. Reactions containing heat denatured enzyme served as controls of specificity. To detect the enzyme activities of CCP5 splicing variants, HEK293 cells were transfected with respective variants harbored in the pCMV-myc vector to generate N-terminal tagged proteins. 40 h after transfection, cells were washed with pre-chilled PBS and lysed in PBS containing 0.2% NP-40. Cell lysate was centrifuged at 40,000 × g at 4 °C for 20 min. 20 μl of the supernatant was incubated with 1 μg porcine tubulin at 37 °C for 5 h. The lysate from cells transfected with myc-tagged LacZ or supplementing 5 mM of the metallocarboxypeptidase inhibitor, 1,10-phenanthroline (OP) to inactivate the enzyme served as controls^42^,^43^. Reactions were terminated with 3x sample buffer and denatured at 95 °C. Subsequently samples were subjected to immunoblot analysis using GT335 (anti-branching point glutamate), polyE (anti-polyglutamate), EP1332Y (anti-α-tubulin), or mouse anti-myc antibodies.

### Rota-rod test

To assess motor coordination, balance, and motor learning, gender- and age-matched littermate mice were tested on rota-rod (San Diego Instruments, San Diego, CA) with an accelerating speed (0 to 40 rpm in 4 min and then hold constant speed for an additional min) as described previously^4^,^26^, and the latency of the mice to fall from the rod was scored as an index of their motor coordination. In the circumstances where only locomotor coordination was assessed, the test was conducted for 1 day. When both locomoter coordination and motor learning were assessed, the test was conducted for 5 consecutive days.

### Statistical methods

The latencies to fall in the rota-rod test were expressed as mean ± SEM (in seconds) and were analyzed for statistical significance using One-way analysis of variance (ANOVA) with repeated measures followed by Bonferroni’s multiple comparison test or Student’s *t*-test for comparison between samples at the same time point. The level of significance was set at *p* < 0.05. In all other experiments, Student’s *t* test was used to compare independent samples for statistical significance. Significance was set at *p* of <0.05. Student’s *t* test was performed using Microsoft^®^ Excel software.

## Additional Information

**How to cite this article**: Wu, H.-Y. *et al*. Role of Cytosolic Carboxypeptidase 5 in Neuronal Survival and Spermatogenesis. *Sci. Rep.* **7**, 41428; doi: 10.1038/srep41428 (2017).

**Publisher's note:** Springer Nature remains neutral with regard to jurisdictional claims in published maps and institutional affiliations.

## Supplementary Material

Supplementary Information
