# Supplementary material for: Role of Cytosolic Carboxypeptidase 5 in Neuronal Survival and Spermatogenesis
Source: Sci Rep. 2017 Jan 27;7:41428. doi: 10.1038/srep41428 (PMC5269731; doi:10.1038/srep41428)
Supplement: Supplementary Information [file srep41428-s1.pdf]

Supplementary Information

Role of Cytosolic Carboxypeptidase 5 in Neuronal Survival and Spermatogenesis

Hui-Yuan Wu[1], Peng Wei[1], James I. Morgan[1]*

[1]Department of Developmental Neurobiology, St. Jude Children's Research Hospital, Memphis, TN, USA

*Corresponding author:

E-mail: jim.morgan@stjude.org (JIM)

Supplementary Figure S1

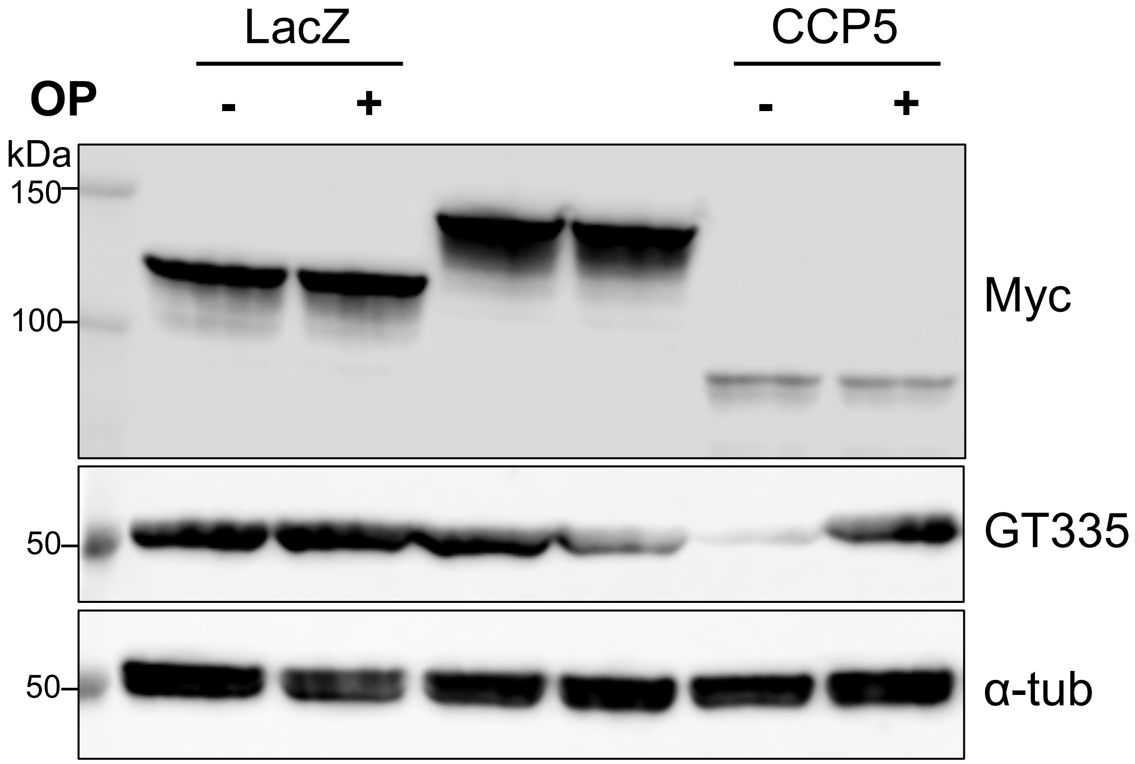

Supplementary Figure S1. The original blots of Fig. 1c, showing that CCP5 activity is specifically inhibited by 1,10-phenanthroline. Those lanes presented in Fig. 1c are indicated. Lanes 3 and 4 are not related to this study and were not presented in Fig. 1c.

Supplementary Figure S2

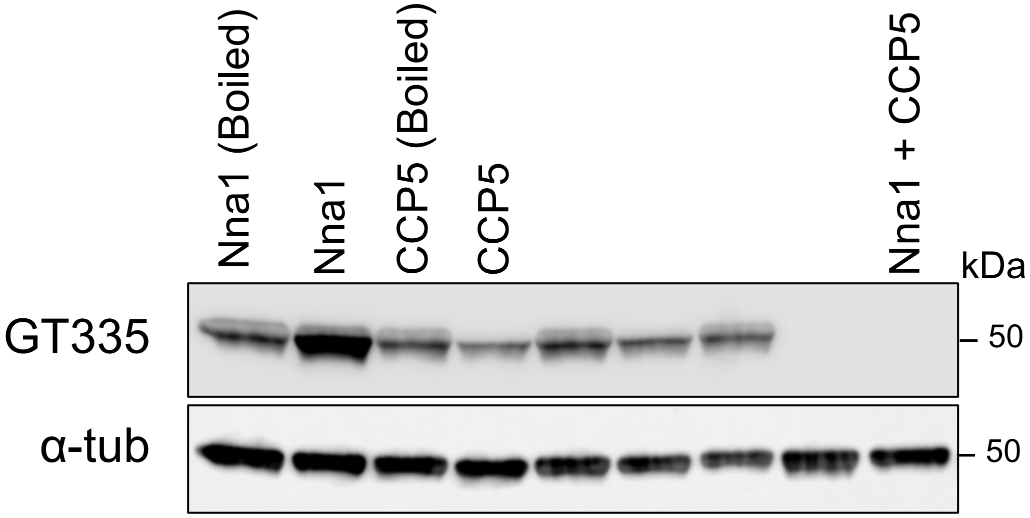

Supplementary Figure S2. The original blots for Fig. 2b, showing the activities of purified recombinant Nna1 and CCP5. The experiments for CCP5 and Nna1+CCP5 were duplicated. For brevity, only designated lanes are presented in Fig. 2b.

Supplementary Figure S3

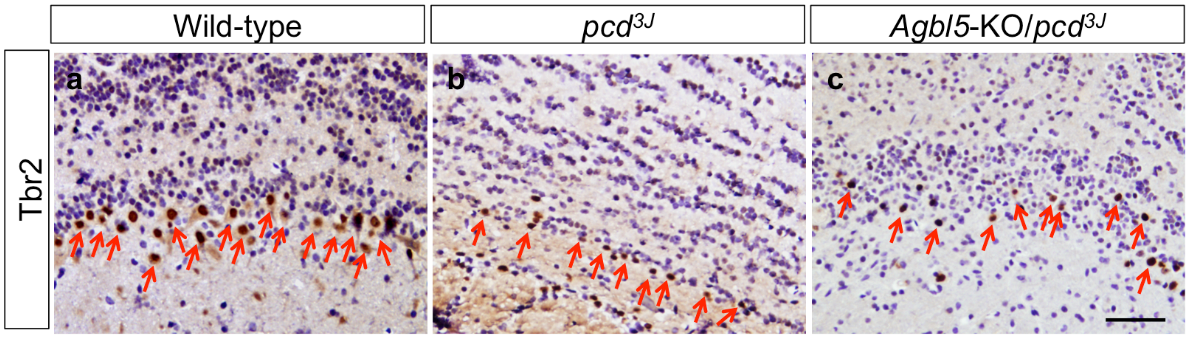

Supplementary Figure S3. Representative images of Tbr2 immunohistochemistry and hematoxylin counterstaining on olfactory bulb sections from 6 months old wild-type (**a**), *pcd*^{3J} (**b**), and *Agbl5*-KO/ *pcd*$^{3J//--}$ double mutant (**c**) mice. While Tbr2 positive olfactory bulb mitral cells (arrows) are preserved in wild-type animals (**a**), there is a similar level of mitral cell loss in *pcd* and *Agbl5*-KO/*pcd*^{3J} double mutants (b, c). Note that the mitral cells in *pcd*^{3J} and *Agbl5*-KO/*pcd*^{3J} olfactory bulbs are shrunk with smaller nuclei than in wild-type mice. Scale bar: 50 µm.

Supplementary Figure S4

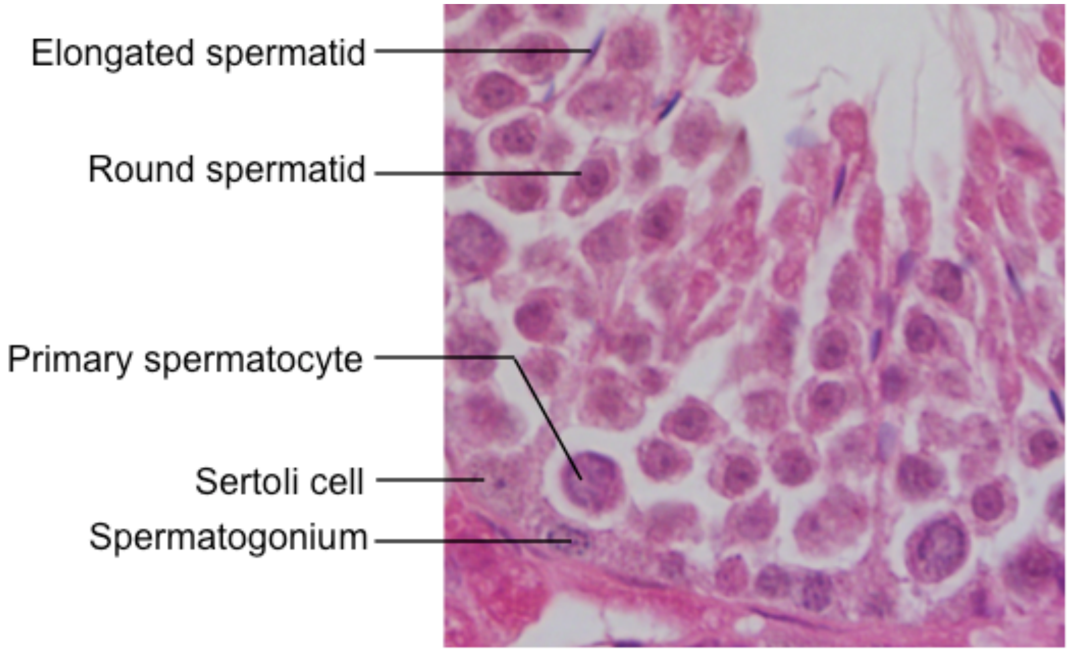

Supplementary Figure S4. Representative image of hematoxylin-eosin staining section of the adult mouse testis. Germ cells of different developmental stages and Sertoli cells are labeled to indicate their typical location in the testis.

Supplementary Figure S5

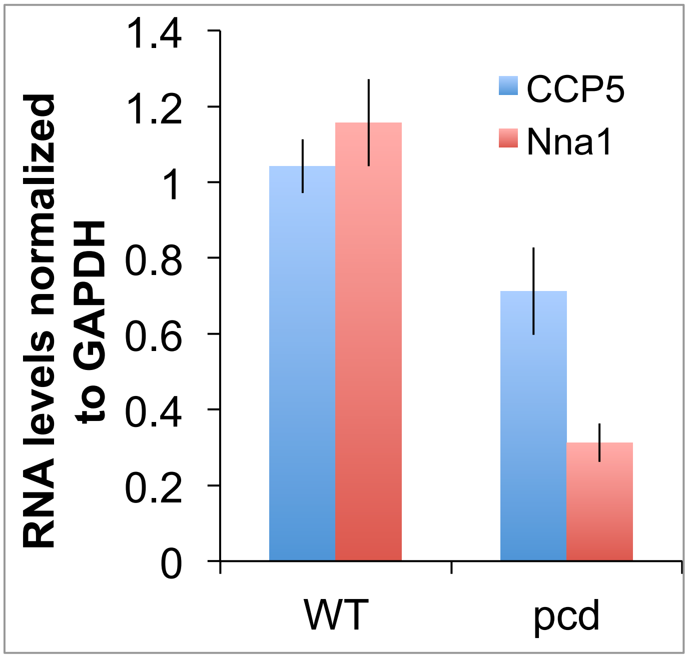

Supplementary Figure S5. Quantitative real-time PCR shows that both *Nna1* and *CCP5* RNA levels are reduced in testis of *pcd* mice compared to that of wild-type littermates at 3-month of age. RNA levels were normalized to internal GAPDH levels and compared with the values of wild-type testis. Bars are mean ± SEM (error bars) of determinations from three animals.
